# The connection between heart rate variability (HRV), neurological health, and cognition: A literature review

**DOI:** 10.3389/fnins.2023.1055445

**Published:** 2023-03-01

**Authors:** Xianghong Arakaki, Rebecca J. Arechavala, Elizabeth H. Choy, Jayveeritz Bautista, Bishop Bliss, Cathleen Molloy, Daw-An Wu, Shinsuke Shimojo, Yang Jiang, Michael T. Kleinman, Robert A. Kloner

**Affiliations:** ^1^Cognition and Brain Integration Laboratory, Department of Neurosciences, Huntington Medical Research Institutes, Pasadena, CA, United States; ^2^Department of Environmental and Occupational Health, University of California, Irvine, Irvine, CA, United States; ^3^Division of Biology and Biological Engineering, California Institute of Technology, Pasadena, CA, United States; ^4^Department of Behavioral Science, College of Medicine, University of Kentucky, Lexington, KY, United States; ^5^Cardiovascular Research, Huntington Medical Research Institutes, Pasadena, CA, United States; ^6^Division of Cardiovascular Medicine, Department of Medicine, Keck School of Medicine of University of Southern California, Los Angeles, CA, United States

**Keywords:** heart rate variability, cognition, inhibitory control, neurological conditions, heart and brain, erratic sinus rhythm, heart rate fragmentation, vagal functioning

## Abstract

The heart and brain have bi-directional influences on each other, including autonomic regulation and hemodynamic connections. Heart rate variability (HRV) measures variation in beat-to-beat intervals. New findings about disorganized sinus rhythm (erratic rhythm, quantified as heart rate fragmentation, HRF) are discussed and suggest overestimation of autonomic activities in HRV changes, especially during aging or cardiovascular events. When excluding HRF, HRV is regulated *via* the central autonomic network (CAN). HRV acts as a proxy of autonomic activity and is associated with executive functions, decision-making, and emotional regulation in our health and wellbeing. Abnormal changes of HRV (e.g., decreased vagal functioning) are observed in various neurological conditions including mild cognitive impairments, dementia, mild traumatic brain injury, migraine, COVID-19, stroke, epilepsy, and psychological conditions (e.g., anxiety, stress, and schizophrenia). Efforts are needed to improve the dynamic and intriguing heart-brain interactions.

## 1. Introduction

**FIGURE 1:**
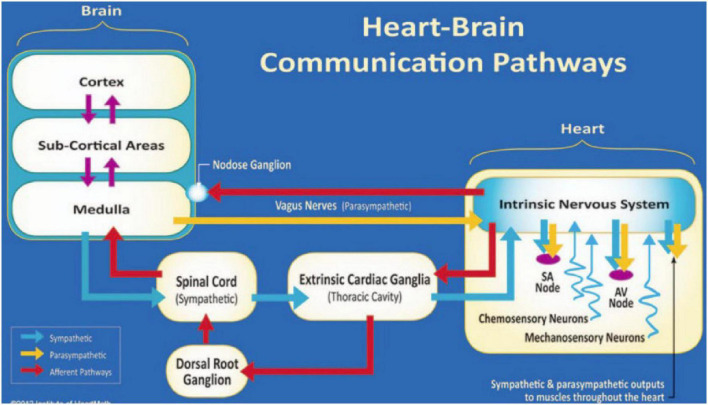
The neural communication pathways interacting between the heart and brain are responsible for the generation of HRV. The intrinsic cardiac nervous system integrates information from the extrinsic nervous system and the sensory neurites within the heart. The extrinsic cardiac ganglia located in the thoracic cavity have connections to the lungs and esophagus and are indirectly connected *via* the spinal cord to many other organs, including the skin and arteries. The vagal nerve (parasympathetic) primarily consists of afferent (flowing to the brain) fibers that connect to the medulla. Used with permission from the Institute of HeartMath, Boulder Creek, California (McCraty and Shaffer, 2015).

The heart and brain connect *via* both electrical and hemodynamic interactions (Figure 1). For example, vagal nerves innervate the heart and mainly target the sinoatrial (SA) node and atrioventricular (AV) node, release acetylcholine upon activation, and decelerate heart rate; while sympathetic nerves project to the SA node, AV node, and most cardiac muscles, release norepinephrine (NE) and epinephrine (E) upon activation, and accelerate heart rate (Levy et al., 1993; Shaffer et al., 2014). The speed at which the molecular signals of each branch are processed influence the speed at which the heart rhythm is affected (Levy et al., 1993). Beat-to-beat variation is more affected by the rapid release and termination of acetylcholine neurotransmission of the vagus nerve. Sympathetic nerves release norepinephrine, which is slower to release and slower to act as it relies on a second messenger in the signaling pathway, therefore affecting the heart rhythm across several beats (Russo et al., 2017). As for signaling from the heart to the brain, over 80% of vagal nerves are afferent and send impulses to the brain (McCraty and Childre, 2010). Sympathetic afferent nerves send autonomic signals to the dorsal root ganglia. Vagal and sympathetic afferent nerves project to both subcortical and cortical regions including brainstem, hypothalamus, thalamus, amygdala, and cerebral cortex (McCraty and Childre, 2010; McCraty and Shaffer, 2015).

Aside from electrical connections, reciprocal hemodynamic connections exist between the heart and the brain. The heart pumps blood which provides oxygen and nutrients to the brain; conversely, deoxygenated blood flows back to the heart from the brain for re-oxygenation through the pulmonary circulation. Reduced cardiac function, as occurs in heart failure secondary to conditions such as myocardial infarction, cardiomyopathy, or valvular heart disease, can result in reduced cerebral blood flow, affecting cognitive function, and contributing to dementia risk (Havakuk et al., 2017; Moore and Jefferson, 2021). Reduction of forward cardiac output due to arrhythmias including chronic atrial fibrillation can reduce cerebral blood flow, leading to cognitive dysfunction (Madhavan et al., 2018). The development of blood clots within the chambers of the heart can contribute to embolic stroke. Atherosclerotic lesions in the aorta and carotid arteries can inhibit blood flow to the brain, and if they dislodge, result in embolic stroke. Hypertension within the vascular system can result in lacunar infarcts to the brain. Thus, a number of cardiovascular hemodynamic conditions can affect cerebral function (Havakuk et al., 2017; Moore and Jefferson, 2021).

These bi-directional electrical and hemodynamic connections between the heart and the brain support physiological, psychological, and cognitive health and influence our physical, mental, and social health (Balconi et al., 2017; Salomon et al., 2017; Forte et al., 2019). For example, stress from natural disasters such as earthquake or a pandemic were associated with increased cardiac death and myocardial infarction (Kloner et al., 1997; Kloner, 2019; Albott et al., 2020; Rollman et al., 2021). Alternatively, heart-brain dysfunction can also be triggered by unnatural factors such as sporting events, when emotionally devoted fans of a Super Bowl game showed increased cardiac death associated with loss by their home teams (Kloner et al., 2009). Another heart-brain dysfunction example concerns the risk of Alzheimer’s disease (AD), where higher Framingham Cardiovascular Risk Score is associated with increased cerebral infarction, cerebral atherosclerosis, and AD pathology (Song et al., 2021).

### 1.1. Heart rate variability (HRV)

Heart rate variability (HRV) is a measure of the variability of beat-to-beat interval (R-R interval) putatively resulting from the dynamic interactions between sympathetic and parasympathetic activities (Prinsloo et al., 2013; Dziembowska et al., 2016). HRV is analyzed from short-term 1 or 5-min electrocardiogram (ECG) recordings of participants at rest and seated in a laboratory setting. Other durations of ECG recordings (long-term such as 24-h) or positions (such as supine) are used based on testing needs. Software used for analysis includes, but is not limited to, Kubios, Biopac, LabChart, or in-house developed codes (such as in MATLAB). The ectopic beats are usually removed before the HRV analysis, since only sinus beats are believed to reflect signals from the brain conducted by autonomic circuits. Recent studies suggested erratic sinus arrhythmia, independent of autonomic regulation, contribute to HRV. These erratic rhythms need to be excluded before autonomic regulation can be interpreted from traditional HRV analysis (Costa et al., 2017, 2018).

#### 1.1.1. Traditional HRV analysis in time and frequency domain

Heart rate variability measurements using linear methods are usually analyzed in the time domain as R-R intervals (RR), heart rate (HR), standard deviation of the normal-to-normal (NN) interval (SDNN), root mean squared successive differences (RMSSD), and the percentage of the number of changes in successive normal sinus (NN) intervals that exceed 50 ms (pNN50). In the frequency domain, HRV measures are analyzed as high frequency (HF), low frequency (LF), very low frequency (VLF), and ultra-low frequency (ULF). Non-linear HRV analysis, including Poincare plots, Power Law Exponent, Approximate Entropy, non-linear prediction, symbolization, phase synchronization, and Detrended Fluctuation Analysis, are less commonly used and are beyond the scope of this review (Francesco et al., 2012; Voss et al., 2015).

Overall, RMSSD and HF reflect vagal inputs to the heart. For example, higher HF, RMSSD, or pNN50 indicates higher vagal functioning, while decreased levels suggest lower vagal activity (Shaffer and Ginsberg, 2017). The ratio of low- and high-frequency power (LF/HF) has been used to estimate the sympatho-vagal balance. However, this concept was disproven by Dr. Billman and others who argued that LF reflected a complex mix of sympathetic, parasympathetic, as well as other unidentified components, and LF/HF was affected by respiration and heart rate independent of autonomic nerve activity (Billman, 2013; von Rosenberg et al., 2017). Therefore, results with symptho-vagal balance interpretation of LF/HF are not further considered in this review.

Standard deviation of the normal-to-normal interval is a marker of total heart rate variability and is influenced more by sympathetic activity than other HRV measures. Additionally, VLF and ULF are usually analyzed from 24-h ECG, and have putatively been linked with circadian rhythm, core body temperature, metabolism, and sleep cycle; but research on VLF and ULF have been limited (Shaffer and Ginsberg, 2017; Table 1). The term “vagal tone” in HRV was derived from previous studies of vagal activity modulation. Examples include direct vagal nerve stimulation induced decreased HRV (Rosenblueth and Simeone, 1934), or increase or decrease in HRV by blockade of autonomic nerve transmission by targeting either the β-adrenergic receptors or the muscarinic receptors, of the sympathetic or parasympathetic systems, respectively (Levy, 1990; Ahmed et al., 1994; Challapalli et al., 1999). We use the term “vagal functioning” instead of “vagal tone” in this review, as additional components of HRV have been reported in the last decades. HRV reflects respiratory activity (respiratory sinus arrhythmia, or RSA) and blood pressure fluctuation (known as Mayer wave ∼0.1 Hz) (Zeng et al., 2018; La Fountaine et al., 2022).

**TABLE 1:** Traditional HRV time-domain and frequency-domain measures and heart rate fragmentation measures.

Parameter	Unit	Description
SDNN	ms	Standard deviation of NN intervals
pNN50	%	Percentage of successive RR intervals that differ by more than 50 ms
RMSSD	ms	Root mean square of successive RR interval differences
ULF power	ms^2^	Absolute power of the ultra-low-frequency band (=0.003 Hz)
VLF power	ms^2^	Absolute power of the very-low-frequency band (0.0033–0.04 Hz)
LF power	ms^2^	Absolute power of the low-frequency band (0.04–0.15 Hz)
LF power	nu	Relative power of the low-frequency band (0.04–0.15 Hz) in normal units
HF power	ms^2^	Absolute power of the high-frequency band (0.15–0.4 Hz)
HF power	nu	Relative power of the high-frequency band (0.15–0.4 Hz) in normal units
LF/HF	%	Ratio of LF-to-HF power
PIP	%	Percentage of inflection points for R-R interval

NN intervals, interbeat intervals from which artifacts have been removed; RR intervals, interbeat intervals between all successive heartbeats. ms, milliseconds; ms^2^, ms squared; nu, normal units; PIP, the percentage of inflection points. Adapted from Tables 1, 2 of Shaffer and Ginsberg (2017) and Costa et al. (2017, 2018).

#### 1.1.2. Heart rate fragmentation (HRF)

Complicated contributors to beat-to-beat variations suggest that the classical HRV as a proxy of sympathetic-parasympathetic balance may be over-interpreted, at least in some situations (Hayano and Yuda, 2019). For example, autonomic regulation is not associated with non-respiratory sinus arrhythmia or erratic sinus rhythm. The erratic rhythm is different from vagal functioning and potentially confounds the prognostic role of traditional time and frequency HRV analysis (Nicolini et al., 2012; Makowiec et al., 2015). Costa et al. have introduced heart rate fragmentation (HRF) analysis, such as percentage of inflection points (PIP) (Table 1), to differentiate between the erratic rhythm and autonomic regulation (Costa et al., 2017, 2018). Erratic sinus rhythm evaluation through non-linear analysis, such as Poincare plots, short-term fractal scaling exponent, is beyond the scope of this review (Stein et al., 2008).

Heart rate fragmentation increased with aging and provided additive value or independent information for classical HRV changes in cardiovascular conditions, such as coronary artery disease or adverse cardiovascular events (Costa et al., 2018; Hayano et al., 2020; Lensen et al., 2020). In the same study of atherosclerosis, Costa et al. (2021) also reported that increased HRF during sleep reflected and predicted decreased global cognitive performance (by Cognitive Abilities Screening Instrument, CASI) and processing speed (by digit symbol coding, DSC).

In this review, although our focus is on RR, HR, RMSSD, SDNN, HF, and LF, it will be important in future studies to clarify the role of HRF vs. autonomic contributions to HRV.

#### 1.1.3. HRV and age, sex, and heart rate

Heart rate variability measures are affected by age and sex. A study of HRV including 1,743 participants ranging from 40 to 100 years old demonstrated that SDNN decreases with age, while RMSSD decreases from 40 to 60 years of age and then increases after 60–70 years of age. Females presented with lower SDNN but with higher RMSSD values than males; and individuals with diabetes presented lower RMSSD and SDNN in both sexes (Almeida-Santos et al., 2016). Another study with over 63,000 participants showed that females presented with higher heart rate and HF (vagal activity) compared to males (Koenig and Thayer, 2016). Voss’s group studied HRV (time domain, frequency domain, and non-linear methods) in 1,906 participants (782 females and 1,124 males) ranging from 25 to 74 years. They reported that in general age influences were stronger than gender influences: SDNN, RMSSD, LF, HF decreased with age in both genders up to 64 years of age. Females (in comparison to males) presented lower LF and increase HF, which disappeared after 44 years (LF) or 54 years of age (HF) (Voss et al., 2015). The idea that RMSSD increases after 60–70 years of age could not only be attributed to vagal functioning, but could also reflect erratic rhythm or increased heart rate fragmentation (Hayano et al., 2020). Thus, HRV studies considering sex difference and age will be helpful in improving HRV reproducibility and replicability, and caution is needed to interpret those changes, especially when studies involve older participants.

Heart rate variability may also be affected by HR, itself. A study of 36 young healthy volunteers inferred that HRV depends more on heart rate (HR) than respiratory rate, and the removal of HR impact improves HRV repeatability (Gasior et al., 2016). However, the clinical significance of the predictive value of HRV can be either dependent or independent of HR. For example, higher HR itself is a risk factor for cognitive decline (Imahori et al., 2021). Consequently, heart rate correction is not routinely used in all forms of HRV analysis.

### 1.2. HRV and brain

**FIGURE 2:**
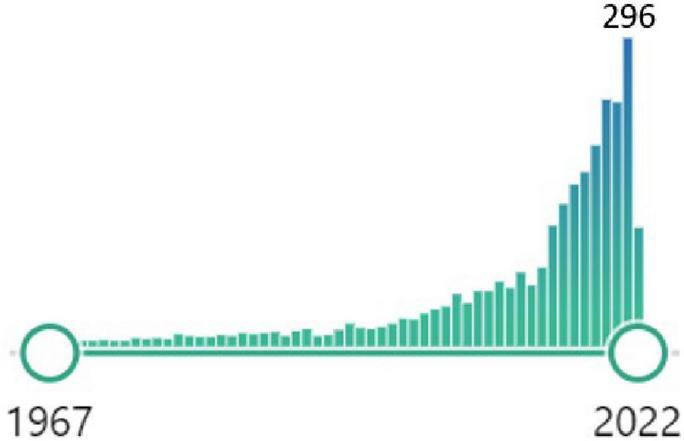
The number of publications on HRV and cognition increases in recent years.

There is increased interest in the link between HRV and cognition (Figure 2). The purpose of this report is to summarize knowledge about links between HRV and cognition, their interactions during physiological state and neurological conditions, potential mechanisms underlying the links, and potentials ways to improve overall health through the links.

## 2. Method

We reviewed the literature on PubMed/Google Scholar database in order to determine the current literature dealing with the link between HRV and cognition. Inclusion criteria were based on key search items, “heart rate variability,” “cognition,” and “dementia” “Alzheimer’s disease,” “traumatic brain injury,” “migraine,” “COVID-19,” “stroke,” “epilepsy,” “psychological conditions,” and “inhibitory control.” Additional search items include “erratic rhythm” and “heart rate fragmentation.” The authors reviewed the citations from review papers involving HRV (linear analysis) and cognition. The final reference list was generated based on relevance to the topics covered in this review. Please note that here we only peek through the lens of “HRV-cognition” to study the heart-brain connection. Due to the complexity of this topic, we may not have included all studies that are relevant to this field.

## 3. HRV changes and neurological status

### 3.1. Physiological conditions

Heart rate variability directly and indirectly affects decision-making related to overall health in our daily life. High HRV (high HF or RMSSD measures) has been linked with greater executive function, better dietary decisions, better controlled social media use, and better negativity avoidance, among others (Allen et al., 2007; Sakaki et al., 2016; Fung et al., 2017; Maier and Hare, 2017; Forte et al., 2019; Williams et al., 2019; Mantantzis et al., 2020).

#### 3.1.1. Executive function

Heart rate variability measures (LF, HF, LF/HF, RMSSD, SDNN) can be potential early markers of cognitive impairment (Forte et al., 2019). In a study of 79 young healthy participants using a 5-min resting ECG analysis, higher resting HF was associated with higher executive function (Williams et al., 2019). Interestingly, lower resting heart rate was correlated with higher executive function, evaluated by composite score averaged from performance in multiple tasks such as color-word interference, visuospatial, and trail making when controlled for lower-order processes such as processing speed (Williams et al., 2019). Additionally, a study including 53 young male sailors showed that higher resting RMSSD (higher vagal functioning) was associated with better working memory (*n*-back of digits) and attention (continuous performance test, CPT), including faster and more accurate responses (Hansen et al., 2003). Inhibitory control has been measured by startle response (e.g., eye blink from auditory stimulation). In an example, 92 college students (Yang et al., 2021) underwent dual-task paradigm that consists of a working memory task (various cognitive loads) and picture viewing task (pleasant, neutral, and unpleasant images), and their resting HRV was measured. Participants with lower resting HF presented a higher startle response magnitude when concurrent working memory load was high, which can result from insufficient top-down resources when the PFC is heavily taxed by high working memory load (Yang et al., 2021). Furthermore, in a study of 50 young healthy participants, Ottaviani et al. have reported positive associations between resting HRV and inhibitory control. In their study, those with higher resting RMSSD predicted better inhibitory control, also after adjustment for confounders including sex, body mass index, and impulsivity (Ottaviani et al., 2019). A path modeling study from a large sample of civil servants in Brazil (*N* = 8,114) has suggested that HF reduction (measured with a 10-min resting-state ECG) was associated with impaired executive function (trail-making B) due to insulin resistance and subclinical atherosclerosis (Kemp et al., 2016).

#### 3.1.2. Decision making

A study of 51 young male participants examined the relationship between resting HRV and dietary decision-making with options of food between healthy nutrients and tasty temptations. Individuals with high HRV presented better resistance to temptation from comfort foods and presented higher ventromedial PFC activity by functional magnetic resonance imaging (fMRI), supporting HRV as a strong dietary self-control biomarker (Maier and Hare, 2017). In that dietary study, SDNN was used as the HRV proxy, as it is a general variability measure which reflects all influences on RR interval series and is known to correlate with measures that reflect phasic vagal activity during resting state (Allen et al., 2007). An additional decision-making example involves resisting social media network overuse. Among 112 participants ranging between 17 and 53 years old, participants exhibiting high impulsivity and low executive function scores tended to suffer from excessive social media use (Wegmann et al., 2020). Interestingly, high HRV were associated with decreased impulsivity (Fung et al., 2017). Therefore, high HRV could directly and indirectly (through better executive function and lower impulsivity) help resist the temptation to social media overuse.

#### 3.1.3. Emotional regulation

Decision-making is influenced by emotional regulation (Martin and Delgado, 2011). A study involving 63 young (mean age of 19) and 62 older (mean age of 72) participants measured resting HRV and eye-tracking while presented with images of faces showing a range of emotions. While young participants, regardless of HRV level, showed no preference between happy or angry faces, older participants with high HRV were more likely to avoid angry faces, suggesting a greater ability to minimize negative influences and a stronger positivity effect that can benefit emotional regulation and health (Mantantzis et al., 2020). Another study of 21 older and 20 young adults used functional MRI (fMRI) and HRV methods. Their results showed that greater resting HRV (RMSSD) was associated with stronger connectivity between amygdala-medial prefrontal cortex (mPFC) across age groups; however, it was associated with stronger connectivity between amygdala-ventrolateral PFC only in the younger group. This study indicates that higher HRV is associated with better emotional regulation across age groups, with some changes in regional associations during aging (Sakaki et al., 2016).

A study including 388 healthy participants of three different age groups suggested that higher RMSSD was linked with higher functional connectivity from bilateral ventromedial PFC in the young adult group and higher functional connectivity from bilateral posterior cingulate cortex across all ages (Kumral et al., 2019). This study supports the role of PFC functional plasticity with aging (Greenwood, 2007).

Therefore, high HRV links with better executive function, decision-making ability, and emotional regulation that benefit health and wellbeing. Further, although cognitive function (PFC connectivity) may change with aging, emotional regulation is resilient to aging.

### 3.2. Pathological conditions: Dementia, traumatic brain injury (TBI), migraine, COVID-19, stroke, epilepsy, and psychological conditions

Studies have linked HRV (ECG analysis) with cognitive dysfunction (including EEG analysis) in dementia, trauma, and COVID-19 (Kim et al., 2006; Nicolini et al., 2014; Purkayastha et al., 2019; Mol et al., 2021), among others.

#### 3.2.1. Neurological conditions

*Dementia* is increasingly a source of great personal, societal, and economic burden. The most common type is Alzheimer’s Disease (AD), followed by vascular dementia, and dementia with Lewy bodies (DLB). Early detection and differentiation as well as the use of simple biomarkers are critical for early prevention as obvious symptoms are lacking until later in disease progression (Nelson et al., 2010). Imbimbo et al. (2022) studied 24-h ECG, European Society of Cardiology Systematic Coronary Risk Evaluation (ESC SCORE), and cognitive function (Montreal Cognitive Assessment, MoCA) in 50 men and women with cardiovascular risk (age ranged 51–77). Their study reported that ultra-low frequency (ULF) HRV was positively associated with MoCA and was negatively associated with ESC SCORE, meaning higher ULF was linked to better cognition and lower ESC SCORE. These findings supported the idea that dysregulation of autonomic nervous system plays an important role in developing cardiovascular risk and cognitive decline (Imbimbo et al., 2022). In a retrospective study of resting HRV on mild cognitive impairment (MCI) patients, compared to the ones who developed AD (MCI-AD, *n* = 23), those who developed DLB (MCI-DLB: *n* = 23) presented lower HRV levels (SDNN, RMSSD, LF, HF), as well as lower visuospatial and frontal executive functions tested by comprehensive neuropsychological test commonly used in Korea (Allan et al., 2007; Kim M. S. et al., 2018). An additional HRV study of 311 elderly women showed that cognitive impairment (Mini-Mental State Examination less than 24) was greater in participants with lower HRV (HF) (Kim et al., 2006). Therefore, lower HRV were observed with cognitive decline.

Nicolini et al. studied HRV of 253 participants [with amnestic MCI (aMCI), non-amnestic MCI (naMCI), or cognitively normal controls], combined with neuropsychological assessment, as well as visual rating scales for hippocampal atrophy (Scheltens’ scale), insular atrophy (Kim’s scale), and cerebrovascular burden (Fazekas’ scale) on brain imaging (Nicolini et al., 2020). Traditionally, it is considered that aMCI is associated with AD pathology, while naMCI is associated with other dementia, such as vascular dementia. Among the three groups, aMCI group presented with blunted normalized low frequency (nLF) increase with postural change (from supine resting to standing): more nLF increase in aMCI related to better episodic memory (prose-delayed recall) and less hippocampal/insula atrophy. This result suggested an overlapping autonomic regulation structure that involves memory processing (Nicolini et al., 2020). On the other hand, naMCI participants presented similar HRV changes to controls: more LF increase related to greater cerebrovascular burden and lower executive function. Their results suggested different autonomic mechanisms between aMCI and naMCI (Nicolini et al., 2020). Another important finding is that HRV changes depend on task types. For example, a cross-sectional study examined 5-min ECGs from 80 older participants with MCI or healthy cognition during supine rest and standing positions (Nicolini et al., 2014). Although no differences in baseline HRV indices between groups were observed, during position changes the MCI participants presented with smaller increases of normalized LF (nLF) and smaller decreases of normalized HF (nHF), indicating reduced physiological changes when transitioning from supine resting to standing (Nicolini et al., 2014). Additionally, Arechavala et al. (2021) used 5-min ECG recordings of 46 cognitively healthy elderly participants classified into two groups (normal or pathological levels of CSF amyloid/tau) at rest or during a computer-based task switching challenge. In this study, there were no differences in baseline HRV between the two groups. However, HRV (RR and LF) decreased from resting to task in those with pathological amyloid/tau, indicating a hyper-active sympathovagal response to a task-switching challenge (Arechavala et al., 2021). These studies suggested that compared to age-matched controls, individuals with early AD risk presented reduced autonomic changes with physical position changes from supine to standing but presented greater responses to mental tasks.

*Mild traumatic brain injury (mTBI) or concussion* is a functional abnormality with reported autonomic dysfunction. A study of 31 young athletes who experienced concussion or mTBI has suggested that HRV (pNN50) decreased only at the acute stage (3 days after injury) and then recovered at around 3 weeks after injury (Purkayastha et al., 2019) compared to 31 undiagnosed young athletes. Higher middle cerebral artery blood velocity at the acute stage was linked with higher HRV (pNN50) and better scores in cognition (Trails making tests) at 3 weeks and 3 month (pNN50) after injury, indicating that insula perfusion (supply by middle cerebral artery) at the acute stage may be one of the underlying predictors for future recovery (Ture et al., 2000; Jordan, 2013; Purkayastha et al., 2019).

*Migraine* has been shown to involve autonomic dysfunction. A cross-sectional study of 36 participants (18 episodic migraine and 18 controls) has shown that migraine patients during ictal stage presented with lower SDNN and LF than controls. The decreased HRV suggested parasympathetic dysfunction that was negatively related to the visual analog scale for pain intensity, meaning lower HRV linked to greater pain intensity (Zhang et al., 2021). Matei et al. (2015) analyzed HRV of 24-h ECG recordings from 27 young patients with migraine (10 with aura and 17 without aura at headache free period) against 10 age-matched healthy controls. They observed consistent autonomic imbalance such as decreased SDNN, RMSSD, and HF in migraine patients (especially those with aura during the night period) supporting parasympathetic hypofunction with sympathetic predominance. Consistent with decreased vagal modulation in migraine, Akter and Ferdousi studied 5-min resting ECG from 60 newly diagnosed migraine patients against 30 age-matched healthy controls. They observed decreased SDNN and RMSSD, as well as higher mean heart rate in the migraine group (Akter and Ferdousi, 2017).

*COVID-19 pandemic* has been a recent global health concern. Results from an online questionnaire and cognitive tests completed by 421 participants reported that memory deficit is associated with fatigue or mixed symptoms (Guo et al., 2022). A retrospective study of 271 hospitalized patients has suggested that higher resting HRV (SDNN) predicted more survival of COVID-19 patients aged 70 and older (Mol et al., 2021). Mol et al. suggested that it was only confirmed in this study that when HRV was low, COVID-19 survival was predicted by age, supporting a protective role of vagal activity in COVID-19 (Mol et al., 2021). Additionally, a study of 17 patients analyzed HRV (SDNN) utilizing 5–7 min daily ECG recordings for a week (Hasty et al., 2020). Results showed that of the 12 patients who developed a C-reactive protein (CRP) surge of 50% or more, 10 were preceded by over 40% drop of HRV (SDNN) within 72 h, and two other patients were treated with convalescent plasma. Therefore, HRV drop predicted an acute inflammation in COVID-19 (Hasty et al., 2020). Another study of 50 participants with a history of COVID-19 infections compared with 50 healthy controls demonstrated decreased HRV in the time domain (such as SDNN and RMSSD) and frequency domain (such as LF and HF), indicating HRV abnormality after COVID-19 (Kurtoglu et al., 2022). Although larger sample sizes are needed for confirmation, these studies suggest that HRV may be a helpful tool in monitoring autonomic dysfunction in COVID-19 patients.

Heart rate variability may help to predict stroke. In part of the Copenhagen Holster Study where 48-h ambulatory ECG and HRV were collected from 678 healthy participants between 55 and 75 years of age, lower nighttime SDNN strongly predicted stroke development even after adjustment for stroke risk factors (Binici et al., 2011). Another study investigated baseline HRV from 5,308 patients that suffered an event of transient ischemic attack (TIA) and minor stroke, followed by functional prognosis and stroke recurrence 90 days after the event. The results suggest that higher SDNN predicts reduced neurological disability and reduced stroke event (Li et al., 2021). In a study of 884 stroke-free Cardiovascular Health Study (CHS) participants, Bodapati et al. examined 24-h ECG-derived HRV and CHS clinical stroke risk score (CHS-SCORE). It was reported that 2 HRV measures, CV% (coefficient of variance of each 5-min NN intervals) and SLOPE (power law slope, or the slope of a line fitted to a plot of log spectral power vs. log of underlying frequency), significantly improved stroke prediction (Bodapati et al., 2017). Therefore, HRV is helpful with stroke detection and prognosis.

*Epilepsy* has been studied with HRV for over 30 years. Patients’ interictal HRV suggests an autonomic balance shift toward sympathetic dominance, which also tends toward further sympathetic overactivity (Myers et al., 2018). In a study of 11 epilepsy patients, HRV was compared from 5-min ECG recordings at 10-5 min and 2 h before seizure onset (Moridani and Farhadi, 2017). Results show that during 5–10 minutes before seizure onset, there were increases of mean HR, LF/HF, and SD2/SD1 (standard deviation of heart rate signals, SD1 shows rapid changes and SD2 describes long-term changes, non-linear analysis in Poincaré plot). Although Poincaré plot analysis is a non-linear method, SD1 and SD2 determined from Poincaré plots are purely linear. These HRV features can potentially be used to define a threshold to aid in predicting seizures (Moridani and Farhadi, 2017). This was supported by a recent study of 238 temporal lobe seizures from 41 patients where HRV features, including decreased RR and pNN50, helped to identify pre-ictal state in 90% of patients and 41% of seizures (Billeci et al., 2018; Leal et al., 2021).

#### 3.2.2. Psychological conditions

Anxiety disorders have been linked with a higher risk of cardiovascular disease. A meta-analysis study of 2,086 patients with anxiety disorder and 2,294 controls free from psychiatric diagnosis suggested that lower HF and time domain measures are associated with anxiety disorders (Chalmers et al., 2014). These results suggest that decreased vagal activity may underlie increased cardiovascular risk in anxiety disorders. *Stress conditions* were reviewed in a meta-analysis of HRV and stress from 37 publications. This overview suggested consistent low vagal activity (decreased HF and increased LF) related to stress (Kim H. G. et al., 2018). *Schizophrenia* as another critical neurological disorder affecting especially younger people, is of great epidemiological importance especially for the healthcare system of developed civilizations (Hasan et al., 2014; Schulz et al., 2020). Schulz et al. (2020) studied cardiorespiratory network coupling for 30 min during rest (NN interval, heart rate, and respiratory frequency) from 23 patients with schizophrenia, 20 first-degree relatives, and 23 healthy controls, using coupling analyses including normalized short-time partial directed coherence, multivariate transfer entropy, and cross conditional entropy. Results suggest compared to controls, schizophrenia patients’ respiration had weaker influences on heart rate, while their heart rate had stronger influences on respiration; their first-degree relatives presented stronger heart rate influences. Results revealed a genetic component of the cardiorespiratory coupling (Schulz et al., 2020). The same group also studied central-autonomic-network (CAN) between central (frontal EEG power), vascular (systolic pressure amplitude), respiratory (frequency), and cardiac (heart rate) activities from 17 schizophrenia patients and 17 controls. Results suggest that schizophrenia patients presented stronger linear respiratory and cardiac influences on central activity, and stronger linear central influences on vascular activity compared to controls (Schulz et al., 2019). Therefore, HRV could be a potential objective measure of psychological distress, including anxiety, stress, and schizophrenia.

Therefore, HRV has been studied in various neurological conditions. HRV analysis may help predict symptoms or outcomes of cognitive decline, migraine, epilepsy, and stroke, and to help identify psychopathologies such as anxiety, depression, and schizophrenia. These changes link pathologies and symptoms such that HRV potentially allows diagnostic and therapeutic supplements in existing pre-clinical and clinical research.

**FIGURE 3:**
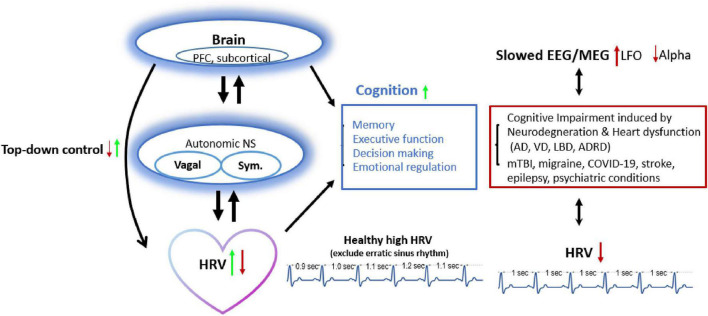
HRV and brain cognition have bidirectional connections: Decreased HRV (from lower top-down inhibitory control) links with reduced executive function, decision making, or emotional regulation and presents in pathological conditions (dementia, mTBI, migraine, etc.). Approach increasing HRV also improves brain activity (e.g., alpha frequency oscillation ranged 8–12 Hz) and top-down control, and vice versa. AD, Alzheimer’s disease; ADRD, Alzheimer’s disease and related dementia; HRV, heart rate variability; LBD, Lewy body dementia; LFO, Low frequency Oscillation; mTBI, mild traumatic brain injury; NS, nerval system; PFC, prefrontal cortex; RIPC, remote ischemic preconditioning; Sym., sympathetic activity; TMS, Transcranial magnetic stimulation; vagal, vagal activity; VD, vascular dementia.

To summarize, high HRV and its associated vagal functioning reflect better executive function, emotional regulation, and decision making in healthy individuals. During pathological condition, HRV was associated with autonomic dysfunction observed in neurological diseases (Figure 3). Efforts are needed to improve our understanding of the heart-brain interactions.

## 4. Updates and limitation

There are some limitations and updates to this topic: (1) We only reviewed literatures using linear analysis of HRV and non-linear HRV analysis were not included. (2) Studies have focused on classical HRV analysis that represent autonomic regulation. However, recent studies have demonstrated that a more disorganized sinus rhythm (erratic rhythm) can contribute to HRV increase in older adults, besides autonomic regulation (Stein et al., 2008; Wdowczyk et al., 2018). The mechanisms underlying the heart rate fragmentation are limited. Details are beyond the scope of this review. Candidate contributors may include cellular, intracellular, extracellular mechanisms, and potential sinus cell uncoupling (Lensen et al., 2020). Therefore, heart rate fragmentation adds to the complexity of what HRV can be interpreted as neuro-autonomic regulation and is also consistent with a limited view of what HRV can tell us. Further studies will be needed to address that. (3) We have reviewed studies that include both short term measures of HRV in the laboratory settings (most studies) and HRV over a prolonged period of time in real life ambulatory settings. With more focused electrophysiological processing, lab settings provide high quality signals and controlled environment, as well as individual’s responses to stimuli (Angius et al., 2019; Arechavala et al., 2021). HRV studies in real life settings have received attention recently with advantages of real-life processing of stimuli. Their challenges include lower signal quality from physical activity and uncontrollable momentary conditions. Several approaches have been used to overcome those challenges for large scale HRV studies, such as additional algorithm for noise reduction, artificial intelligence, and use appropriate reference state (Smets et al., 2019). For example, carefully designed experiments are needed with data collected at different times or conditions for the same individuals that allow self-controlled data (Smets et al., 2019). Further, commercial technologies provide wearable sensors for real life HRV studies, which have supported their application for objective measures of stress or relaxed state and orthostatic challenge (Hernando et al., 2018; Gambassi et al., 2020). Interestingly, HRV study during a simulated virtual environment has been shown to detect stress-vulnerable individuals (Rodrigues et al., 2020). (4) HRV measured under real life or free-running conditions needs to be carefully interpreted. The information at the time of measurement is often insufficient, and it is difficult to tell whether the HRV analyses reflect characteristics of autonomic function or indirectly reflect characteristics of behavior or psychophysical activity. For example, a lower LF/HF during free activity is associated with worse life expectancy (Shimizu et al., 2002). However, when people spend more time in the supine position during the day, they may have a lower mean LF/HF, but it is not associated with lower sympathetic activity (lower stress or better cardiac function).

## 5. Conclusion

The heart and brain have bi-directional influences on each other, and both are involved in an individual’s proper reactions to internal and environmental signals. The reciprocal support and regulation help wellbeing while dysregulations (e.g., decreased vagal functioning or increased HRF) can be associated with cognitive dysfunction or other neurological conditions.

## Author contributions

XA, MK, and RK: conception and design of the review. XA, RA, EC, JB, BB, CM, and D-AW: acquisition of the materials. XA and RA: writing the manuscript. XA, RA, EC, JB, BB, CM, D-AW, SS, YJ, MK, and RK: editing the manuscript. All authors contributed toward discussions of the review and the final manuscript.
